# Fisetin Alleviated Bleomycin-Induced Pulmonary Fibrosis Partly by Rescuing Alveolar Epithelial Cells From Senescence

**DOI:** 10.3389/fphar.2020.553690

**Published:** 2020-12-14

**Authors:** Li Zhang, Xiang Tong, Jizhen Huang, Man Wu, Shijie Zhang, Dongguang Wang, SiTong Liu, Hong Fan

**Affiliations:** Department of Respiratory and Critical Care Medicine, West China Hospital/West China School of Medicine, Chengdu, China

**Keywords:** fisetin, pulmonary fibrosis, cellular senescence, senescence-associated secretory phenotype, AMP-activated protein kinase

## Abstract

Idiopathic pulmonary fibrosis is an aging-associated disease, satisfactory therapies are not yet available. Accelerated senescence of alveolar epithelial cells plays an important part in Idiopathic pulmonary fibrosis pathogenesis. Fisetin (FIS) is a natural non-toxic flavonoid, which has many pharmacological functions. However, the role of FIS in pulmonary fibrosis has not been established. In this study, we found that FIS treatment apparently alleviated BLM-induced weight loss, inflammatory cells infiltration, inflammatory factors expression, collagen deposition and alveolar epithelial cell senescence, along with AMPK activation and the down regulation of NF-κB and TGF-β/Smad3 *in vivo*. *In vitro*, FIS administration significantly inhibited the senescence of alveolar epithelial cells and senescence-associated secretory phenotype, followed by reduced transdifferentiation of fibroblasts to myofibroblasts as well as collagen deposition in fibroblasts, which was blocked by an AMPK inhibitor, Compound C. Together, these results suggest that FIS can alleviate the development of BLM-induced pulmonary fibrosis, which is related to the inhibition of TGF-β/Smad3 signaling and the reduction of alveolar epithelium cell senescence by regulating AMPK/NF-κB signaling pathway. FIS may be a promising candidate for patients with pulmonary fibrosis.

## Introduction

Idiopathic pulmonary fibrosis (IPF) is a chronic, progressive lung disease, which is characterized by the aberrant accumulation of extracellular matrix (ECM) in the lung parenchyma and deterioration of lung function (Richeldi et al., 2017). Both clinical observations and epidemiological investigations indicate that IPF is an aging-associated disease, since IPF occurs primarily in middle-aged and elderly people (median age at diagnosis is around 65 years), and the incidence rises remarkably with advancing age (Richeldi et al., 2017; Tian et al., 2019). More importantly, all nine primary markers of aging have been found to participate in the pathogenesis of IPF (López-Otín et al., 2013; Meiners et al., 2015). Several scholars even use the term “senescence-associated pulmonary fibrosis” to describe the phenomenon (Chen et al., 2020).

Cellular senescence is pivotal for phenotype of aging (Meiners et al., 2015). The characteristics of senescent cells include growth arrest, enlarged cell morphology, elevated activity of SA-β-Gal as well as increased expression of cell cycle inhibitors, such as p16 and p21 (Kuwano et al., 2016). Dysfunctional re-epithelialisation, following repetitive micro-injury, initiates the process of pulmonary fibrosis (PF). Increasing evidences have implicated that accelerated senescence of alveolar epithelial cells, a main cause of epithelial dysfunction, plays an important role in IPF pathogenesis (Kuwano et al., 2016; Lehmann et al., 2017). Senescent alveolar epithelial cells not only lose the ability of regeneration and repair, but also exert deleterious effects on neighboring cells by secreting a variety of proinflammatory cytokines, pro-fibrosis factors, growth factors, matrix metalloproteinases and chemokines, described as senescence-associated secretory phenotype (SASP) (Salminen et al., 2012).

Fisetin (FIS), a natural non-toxic flavonoid, is present in various plants, fruits and vegetables. Previous researches have demonstrated that FIS has anti-inflammatory (Huang et al., 2018), anti-fibrosis, anti-oxidant (Hussain et al., 2019) and anti-aging properties (Yousefzadeh et al., 2018). FIS supplementation can prevent or alleviate hepatic and myocardial fibrosis by inhibiting the expression of fibrosis-related genes and inactivating TGF-β1/Smad3 signaling (Liu et al., 2019; Choi et al., 2020; Hu et al., 2020). Senolytic drugs, dasatinib and quercetin (D + Q), can attenuate experimental PF via selective depletion of senescent alveolar epithelial cells (Lehmann et al., 2017). More encouragingly, a recent first-in-human open-label clinical trial has suggested that short-term administration of D + Q could improve the physical dysfunction in IPF patients (Justice et al., 2019). Both FIS and quercetin belong to the flavonoid class, and FIS exhibits stronger senotherapeutic activity in cultured cells than quercetin, and can extend lifespan in mice (Yousefzadeh et al., 2018). These traits remind us that FIS may have protective effect in PF. However, the role of FIS in PF has not been elucidated.

AMP-activated kinase (AMPK), a dominant regulator of energy metabolism, not only controls the aging process, but also regulates the development of lung fibrosis (Salminen and Kaarniranta, 2012; Jiang et al., 2017). Currently, AMPK is viewed as a potential therapeutic target for PF. Bleomycin (BLM)-induced PF is the most frequently used animal model. Treatment with BLM can also induce alveolar epithelial cell senescence *in vitro* and *in vivo* (Aoshiba et al., 2003; Qiu et al., 2019). In this study, BLM was used to reproduce PF in mice and induce alveolar epithelial cell senescence to investigate the effect and mechanism of FIS in experimental PF.

## Materials and Methods

### Chemicals and Reagents

FIS (purity over 98%), Compound C (CC) and BLM were purchased from Selleck (Houston, United States). Hydroxyproline (HYP) Assay Kit was obtained from Nanjing Jiancheng Bio-Engineering Institute (Nanjing, China). Antibodies against GAPDH, AMPK, phospho-AMPK (p-AMPK), Smad3, phospho-Smad3 (p-Smad3), p65, and phospho-P65 were provided by Cell Signal Technology, Inc. (Massachusetts, United States). Antibodies against *β*-actin, TGF-β and alpha-smooth muscle actin (α-SMA) were purchased from Proteintech Group, Inc. (Wuhan, China). Antibodies against Collagen 1, p21 and p16 were gained from Abcam Technology Inc (Cambridge, United Kingdom).

### Animals

Male C57BL/6J mice (25 ± 2 g), 8–10 weeks of age, were supplied by GemPharmatech Co. Ltd. (Jiangsu, China). All animals for this experiment were fed with standard diet and water at libitum and housed in a temperature-controlled room at 23 ± 2 °C and 50–60% humidity (12 h light/dark cycle) for 1 week before the experiments. All animals received care in accordance with the recommendations of the National Institutes of Health Guide for Care and Use of Laboratory Animals, and the experiment scheme was approved by the Committee on the Ethics of Animal Experiments of Sichuan University.

### Mouse Model of Bleomycin-Induced Pulmonary Fibrosis

The animal model of BLM-induced pulmonary fibrosis was performed as described previously (Aoshiba et al., 2013). In brief, 70 mice were randomly divided into four groups. Control (CON) group: mice were given intra-tracheal administration of 50 μl of saline at day 0; FIS group: the saline-instilled mice were intragastrically administered with 100 mg/kg of FIS (suspended in 150 μl of 0.5% carboxy methyl cellulose sodium) starting at day 7 and treated once every other day for 3 weeks (Yousefzadeh et al., 2018); BLM group: mice were given intra-tracheal instillation of 50 μl of bleomycin (2.5 mg/kg); BLM + FIS group: On day 7 after BLM treatment, mice were orally administered with FIS once every other day for 3 weeks. On day 28, mice were euthanized by intraperitoneal injection of excessive pentobarbital sodium.

### Broncho-Alveolar Lavage Fluid Collection

The BALF was harvested according to previously described (Wang et al., 2018). Briefly, the right lung was lavaged with 0.5 ml of iced phosphate-buffered saline (PBS) for three times, and fluid recovery is about 80% per time. The BALF were centrifuged at 1,000 g for 5 min, and then the supernatant was collected and stored at −80 °C.

### Histological Analysis and Immunohistochemistry

The left lung tissues were fixed with 4% formalin, embedded in paraffin. Histological sections (6 μm) were stained with hematoxylin-eosin (HE) and Masson’s trichrome staining. According to previous literature, the dimensions of inflammation and pulmonary fibrosis were evaluated quantitatively (Szapiel et al., 1979). Immunohistochemistry was used to investigate the expression of p21. Paraffin-embedded lung sections were dewaxed with xylene, and endogenous peroxidase was removed with 3% H_2_O_2_. Antigen retrieval was performed by heating tissue sections, and non-specific binding sites were blocked with 5% BSA for 20 min. The primary anti-p21 antibody (1:200) was incubated at 4°C overnight, and the second antibody was incubated at room temperature for 1 h. Color was visualized via incubation with diaminobenzidine.

### Hydroxyproline Assay

HYP content in lung tissues was determined by using a test kit according to the manufacturers’ instructions. About 30 mg of tissue samples were mixed with 1 ml of hydrolysase, placed in 60°C boiling water bath for about 20 min. After centrifugation of the supernatant at 3,500 rpm/min for 10 min, the absorbance at 550 nm was determined. HYP content was expressed as μg/mg.

### Cell Culture

A549 and human embryonic lung fibroblast (HELF) cells were purchased from American Type Culture Collection (Rockville, MD, United States). These cells were cultured in DMEM (Hyclone, United States) supplemented with 10% FBS (ZETA, United States) and 1× solution of penicillin-streptomycin (Hyclone) at 37°C in a humidified 5% CO_2_ atmosphere.

To build the model of alveolar epithelial cell senescence and select the optimal concentration of BLM, A549 cells were stimulated with different doses of BLM for 3 days (Qiu et al., 2019). Next, we observed the roles of FIS and AMPK in alveolar epithelial cell senescence. Before BLM (5 μg/ml) was added to induce cell senescence, A549 cells in six-well plate were pretreated with or without 10 μM Compound C (CC, an inhibitor of AMPK) for 1 h (Liu et al., 2018). Subsequently, cells grew in the presence or absence of FIS (10 μM) for 3 days (Yousefzadeh et al., 2018).

In order to mimic SASP and observe its effects on epithelial-mesenchymal crosstalk, we used A549-derived conditioned mediums to further culture HELF cells. After treating with or without 5 μg/ml of BLM and 10 μM FIS for 3 days, the culture supernatants of A549 cells were replaced by fresh culture mediums without BLM, and A549 cells were cultured for another 2 days. Normal A549-derived conditioned mediums (N-CM), BLM-induced A549-derived conditioned mediums (B-CM) as well as BLM and FIS co-stimulated A549-derived conditioned mediums (BF-CM) were collected to analyze cytokines and further culture HELF cells with one half of concentration for 3 days. The protein expressions of collagen 1 and α-SMA in HELF cells were detected to reflect the fibrotic response.

### Senescence-Associated β-Galactosidase Staining

SA-β-Gal staining was performed by using the senescence *β*-Galactosidase staining kit (Beyotime, Shanghai, China) as previously described (Tian et al., 2019). Frozen lung tissue sections and cell samples were fixed with 4% formaldehyde for 10–15 min at room temperature, washed three times with PBS for 5 min each time, and then placed in freshly prepared staining solution overnight at 37°C. To better show lung structure, the tissue sections were further stained with eosin. SA-β-Gal-positive cells (bluish green color) were counted in three random microscopic fields and expressed as % of total cells.

### Western Blot Analysis

Lung tissues or cells were collected and lysed in ice-cold RIPA lysis buffer containing fresh protease and phosphatase inhibitor cocktails (MedChemExpress, United States). Protein samples were separated on 10% or 12.5% SDS-PAGE gels, and then transferred to methanol-activated PVDF membranes (Merck Millipore, Germany). After 1 h blocking with 5% nonfat milk, these membranes were incubated with primary antibodies overnight at 4°C. After membranes were washed three times, the corresponding secondary antibody was added for incubation at room temperature for 1–2 h. Protein expression was visualized using ECL (GE Healthcare, United Kingdom), and band intensities were measured using ImageJ software 6.0.

### Real-Time Quantitative Polymerase Chain Reaction Assay

Total RNA was extracted from lung tissues using TRIzol reagent (Invitrogen Life Technologies, United States), reverse-transcribed to complementary DNA (cDNA) using ReverTra Ace qPCR RT Master Mix with gDNA Remover (Toyobo, Japan). Relative gene expression was quantified using iTaq Universal SYBR Green Supermix (Bio-Rad, United States). Table 1 shows the primer sequences, relative gene expression levels were normalized to β-actin and calculated using the 2^−ΔΔCt^ method.

**TABLE 1:** Primers for quantitative real-time (qRT)-PCR.

Primer name	Sequence (5' to 3')
Mouse p16-F	ATCTGGAGCAGCATGGAGTC
Mouse p16-R	TCGAATCTGCACCGTAGTTG
Mouse p21-F	CCTGGTGATGTCCGACCTG
Mouse p21-R	CCATGAGCGCATCGCAATC
Mouse collagen I-F	CTGTCTGCTTCCTGTAAACT
Mouse collagen I-R	TCCATGTGAAATTGTCTCCC
Mouse *α*-SMA-F	TCAGGGAGTAATGGTTGGAATG
Mouse *α*-SMA-R	CCAGAGTCCAGCACAATACCAG
Mouse *β*-actin-F	CACTCTTCCAGCCTTCCTTCC
Mouse *β*-actin-R	AGGTCTTTGCGGATGTCCAC

### Enzyme-Linked Immunosorbent Assay Detection

The levels of IL-1β, IL-6, TGF-β, MMP-9, and TNF-α in BALF and culture mediums were detected by ELISA assay following the manufacturer’s instructions (NeoBioscience, Shenzhen, China).

### Statistical Analysis

Statistical analyses were performed using GraphPad Prism version 6.0 (GraphPad software, United States). All the original data were showed as mean ± SD. One-way analysis of variance and repeated-measures analysis of variance followed by the Tukey’s multiple comparison test were used for comparisons of multiple groups. The survival curve of each group was drawn using the Kaplan-Meier method. Values of *p* < 0.05 were considered statistically significant.

## Results

### Fisetin Alleviated Bleomycin-Induced Pulmonary Fibrosis in Mice

**FIGURE 1:**
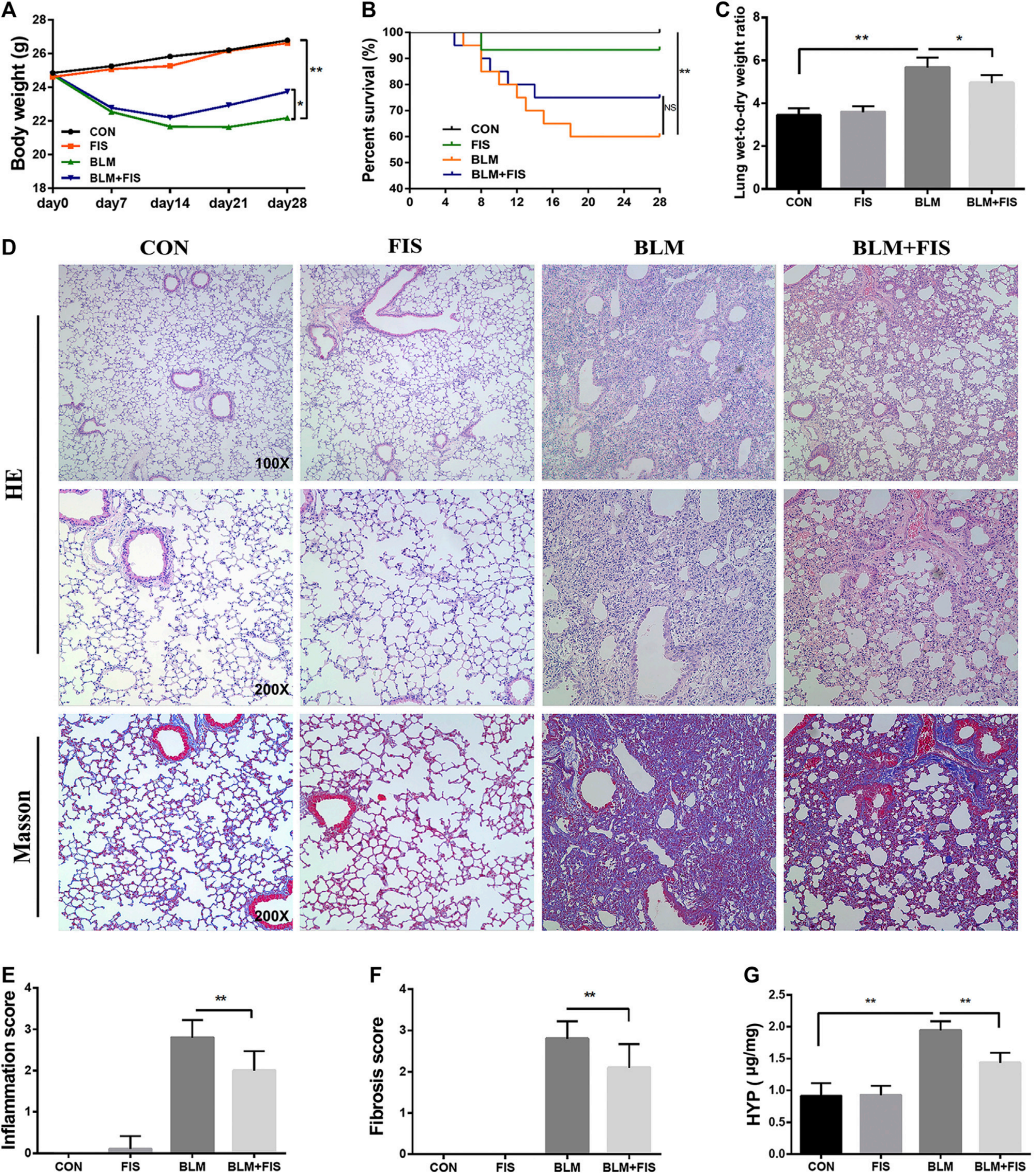
Fisetin (FIS) alleviated bleomycin (BLM)-induced pulmonary fibrosis in mice. On day 7 after BLM treatment (2.5 mg/kg), mice were intragastrically administered with FIS (100 mg/kg) once every other day for 3 weeks. Mouse lungs were collected on day 28 after BLM treatment. The body weight **(A)**, survival rate **(B)**, lung wet-to-dry weight ratio **(C)**, and hydroxyproline content **(G)** in lungs were determined. The representative images of HE-stained (×100 and ×200 magnification) and Masson’s trichrome-stained (×200 magnification) lung sections in mice **(D)** and comparisons of the inflammation score **(E)** and fibrosis score **(F)** between the experimental groups were shown. Data were presented as the means ± SD. ^*^ *p* < 0.05, ^**^ *p* < 0.01. NS, non-significant.

After intra-tracheal instillation of BLM, significant weight loss, survival rate reduction and increased wet/dry weight ratio of lung were noticed (Figures 1A–C). The seventh day after BLM administration is considered to be the beginning of the fibrotic phase (Li et al., 2019), FIS was intragastrically administered at day 7 for 21 days. BLM-induced weight loss and the increase in the wet/dry weight ratio were attenuated by FIS administration. There was no significant difference in the survival rate between the BLM group and BLM + FIS group, but a trend was revealed that FIS treatment would improve the survival rate of mice (Figure 1B).

In terms of histopathology, no significant pathological alteration in lung architecture was observed in the CON and FIS group. However, HE staining showed obvious morphologic changes of lung in the BLM group, including thickening of alveolar septum, alveolar structure destruction, and heavy inflammatory cell infiltration in alveolar space and pulmonary interstitium (Figure 1D). In addition, the lung tissues of BLM treated mice also displayed an increase in collagen deposition, which was evident from the increase of blue fiber bundles in Masson-stained lung sections (Figure 1D). As expected, treatment with FIS markedly reduced inflammatory cell infiltration, interstitial thickness and collagen deposition. In accordance with these observations, there was a statistically significant increase in the inflammation and fibrosis scores in the BLM group, while protection of FIS on lung microstructure was clearly reflected in the lower scores in the BLM + FIS group (Figures 1E,F).

**FIGURE 2:**
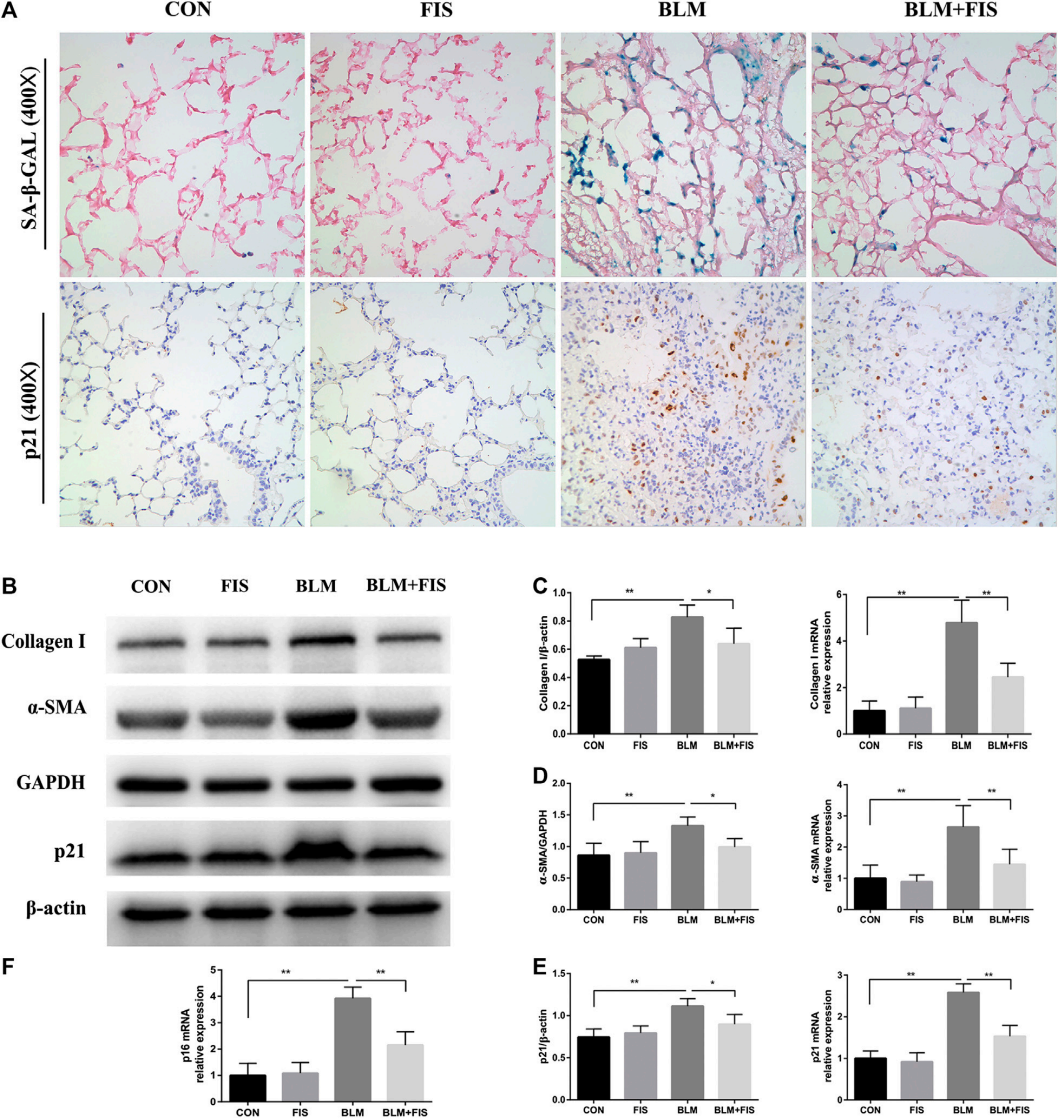
FIS reduced the senescent cell burden and attenuated fibrotic marker in fibrotic lung tissue of mice. One week after 2.5 mg/kg of BLM treatment, mice were orally administered with FIS (100 mg/kg) once every other day for 3 weeks. Representative images of SA‐β‐Gal staining and immunohistochemistry for p21 were displayed under ×400 magnification **(A)**. Protein and gene expression of collagen 1 **(B, C)**, *α*-SMA **(B, D)** and p21 **(B, E)** as well as p16 mRNA level **(F)** were detected using Western blot and quantitative real-time (qRT)-PCR. Data were expressed as mean ± SD (*n* ≥ 3). ^*^ *p* < 0.05, ^**^ *p* < 0.01.

Massive collagen secreted by myofibroblasts is the major source of ECM, while *α*-SMA is a biomarker protein of myofibroblasts (Li et al., 2019). HYP is one of the main components of collagen. As shown in Figures 2B–D, FIS administration obviously reduced the expressions of collagen 1 and *α*-SMA both at mRNA and protein levels as well as HYP content (Figure 1G) in lung tissues, suggesting that FIS suppressed transdifferentiation of fibroblasts to myofibroblasts and therefore decreased accumulation of collagen. Taken together, these data indicate that FIS can effectively alleviate the development of BLM-induced PF.

### Fisetin Attenuated Bleomycin-Induced Alveolar Epithelial Cell Senescence and Inflammatory Responses *in Vivo*

Prior studies have shown that BLM treatment can effectively induce cellular senescence in alveolar epithelial cells (Aoshiba et al., 2003). Cyclin-dependent kinase inhibitors p16 and p21, as well as SA-β-Gal activity are often used to confirm the appearance of senescence (Aoshiba et al., 2013; Qiu et al., 2019). As shown in Figure 2, both p16 and p21 gene expressions were apparently up-regulated in BLM-induced fibrotic mouse lungs, p21 protein was also significantly increased compared with the control group. Unfortunately, due to lacking of an ideal p16 antibody for mice (Demaria et al., 2014; Lehmann et al., 2017), we could not analyze p16 on protein level. In addition, using immunohistochemical staining, we found that the positive staining of p21 was mainly located in lung structural cells that morphologically resembled alveolar epithelial cells in BLM-induced mice (Figure 2A). Subsequently, we evaluated SA‐β‐Gal activity, a widely used indicator for detecting senescent cells, which will be dyed bluish green color. As previous findings demonstrated, an increase in the number of blue-green stained senescent cells was observed in fibrotic lung tissues, whereas that was barely seen in normal lungs (Figure 2A). However, both the strong SA-β-gal positive staining and the elevated expression of p21 and p16 induced by BLM were significantly alleviated by treatment with FIS compared to the BLM group.

**FIGURE 3:**
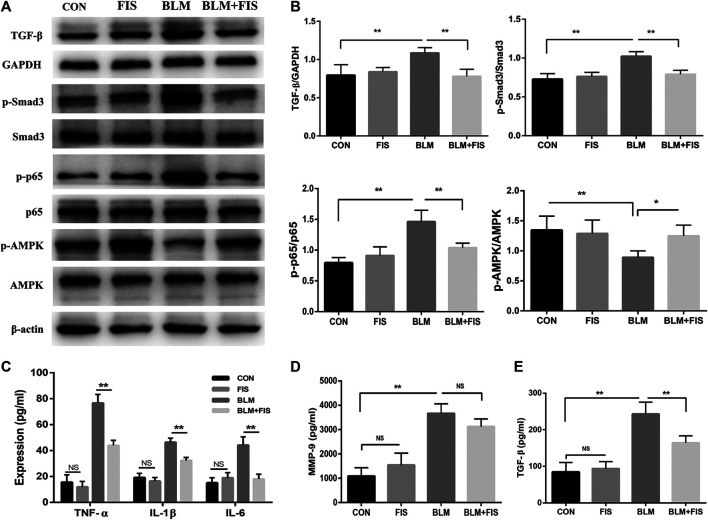
FIS regulated the activity of AMPK and NF-κB and inflammatory responses in BLM-induced mice. One week after 2.5 mg/kg of BLM treatment, mice were orally administered with FIS (100 mg/kg) once every other day for 3 weeks. Protein expressions of TGF-β, p-Smad3/Smad3, p-AMPK/AMPK and p-p65/p65 in lung tissue were detected by Western blotting analysis **(A, B)**. The levels of IL-1β, IL-6, TGF-β, MMP-9 and TNF-α in BALF were detected by ELISA assay. Data were expressed as mean ± SD (*n* ≥ 3). ^*^ *p* < 0.05, ^**^ *p* < 0.01. NS, non-significant.

To further explore the effects of FIS on inflammatory reaction, several common cytokines were analyzed by ELISA, which have also been suggested to be part of the SASP (Aoshiba et al., 2013; Qiu et al., 2019). In Figure 3, the levels of pro-inflammatory cytokines (IL-1β, IL-6, TNF-α), profibrogenic cytokine (TGF-β) and metalloproteinase-9 (MMP-9) in the BALF of BLM-induced mice were significantly higher than those in healthy mice. In contrast, intervention with FIS dramatically decreased the release of these cytokines, except for MMP-9. Together, these results indicate that FIS can attenuate BLM-induced alveolar epithelial cells senescence and inflammatory responses *in vivo*.

### Fisetin Inhibited Transforming Growth Factor-β/Smad3 Signaling and Regulated the Activity of AMP-Activated Protein Kinase and Nuclear Factor-κB in Pulmonary Fibrosis in Mice

TGF-β/Smad3 signaling plays a critical role in the development of pulmonary fibrosis. The phosphorylation of Smad3 was used to reflect Smad3 activity. As shown in Figures 3A,B, compared with the CON and FIS group, the protein levels of TGF-β and phospho-Smad3 (p-Smad3) were significantly increased in the BLM group. However, the increase was effectively reduced in the BLM + FIS group.

The activity of AMPK were evaluated by measuring the phosphorylation of Thr172 of AMPK. As illustrated in Figure 3, the levels of phosphorylated AMPK (p-AMPK) were obviously lower in the BLM group as compared to the CON and FIS group. However, FIS evidently increased the expression of p-AMPK thus to enhance AMPK activity in lungs compared with the BLM group.

Similarly, we detected the phosphorylation levels of p65 component of NF-κB to reflect NF-κB activity. Elevated levels of phospho-p65 (p-p65) were detected in the BLM group as compared to the CON and FIS group, while p-p65 protein levels in the BLM + FIS group were obviously lower than that in the BLM group (Figures 3A,B). These results suggest that FIS can suppress TGF-β/Smad3 signaling and regulate the activity of AMPK and NF-κB.

### Fisetin Relieved Bleomycin-Mediated Alveolar Epithelial Cell Senescence via AMP-Activated Protein Kinase/Nuclear Factor-κB Signaling Pathway *in Vitro*

**FIGURE 4:**
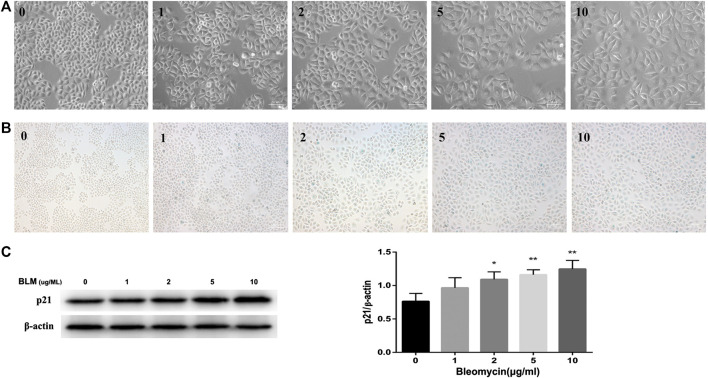
BLM induced senescence in A549 cells in a dose-dependent manner. A549 cells were stimulated with different doses of BLM for 3 days. Morphology of A549 cells treated with 0–10 μg/ml of BLM was observed under ×200 magnification **(A)**. SA‐β‐Gal staining was shown under ×100 magnification **(B)**. P21 protein expression was detected by Western blotting analysis **(C)**. ^*^ *p* < 0.05 and ^**^ *p* < 0.01 is significantly different from the control.

Refer to previous literatures (Aoshiba et al., 2003; Qiu et al., 2019; Tian et al., 2019), we used BLM to build a cellular senescence model. Different concentrations of BLM were used to stimulate A549 cells for 3 days. Treatment of A549 cells with BLM caused a dose-dependent increase in protein expression of p21 (Figure 4C), and this increase paralleled with a distinct, flat, and enlarged cell morphology (Figure 4A). Meanwhile, with the increase of BLM concentration, the SA‐β‐Gal activity was also up-regulated. However, there was no significant change between 5 and 10 μg/ml of BLM (Figure 4B). Therefore, the following experiments were continued with 5 μg/ml of BLM.

**FIGURE 5:**
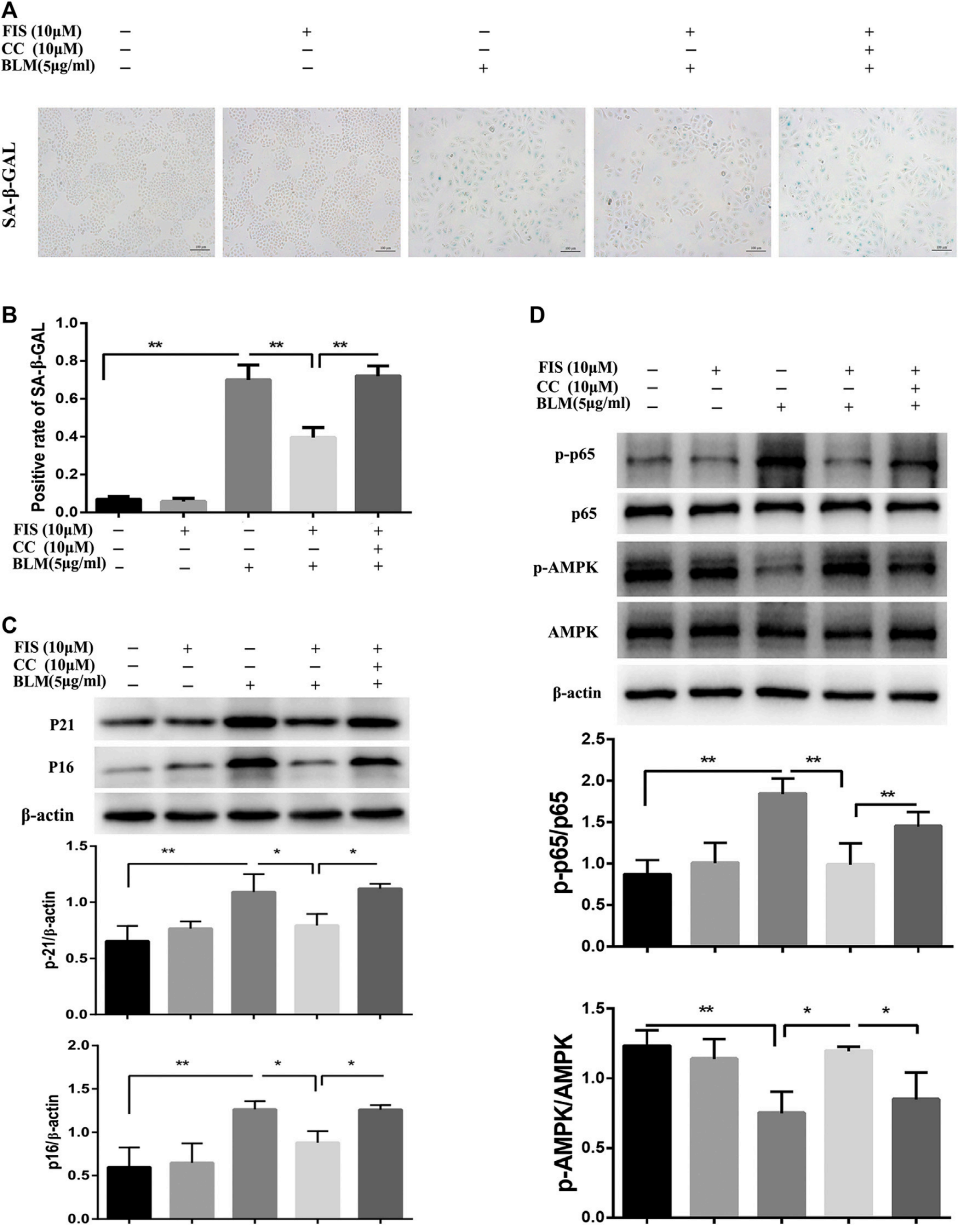
FIS relieved BLM-mediated senescence in A549 cells via regulating AMPK/NF-κB signaling pathway. A549 cells were pretreated with 10 μM Compound C (CC) and subsequently incubated with/without BLM (5 μg/ml), FIS (10 μM) for 3 days. SA‐β‐Gal staining was shown under ×100 magnification **(A, B)**. Protein expressions of p16, p21, p-AMPK/AMPK and p-p65/P65 were detected by Western blotting analysis **(C, D)**. Data were shown as mean ± SD (*n* ≥ 3). ^*^ *p* < 0.05, ^**^ *p* < 0.01.

Further study found that the activity of SA‐β‐Gal in BLM-induced senescent A549 cells was remarkably increased as compared to the CON and FIS group, while FIS prevented this change (Figures 5A,B). Consistent with the results of *in vivo* experiments, the decreased protein expressions of p-AMPK and increased protein levels of p-p65 were detected in BLM-induced senescent A549 cells (Figure 5D), which were obviously improved by FIS, whereas such changes were reversed by AMPK inhibitor Compound C (CC). Similarly, FIS notably inhibited p21 and p16 protein expressions, which were partially blocked by CC (Figure 5C). These findings confirme that FIS can effectively relieve alveolar epithelial cell senescence partly through activating AMPK and inhibiting NF-κB activity.

### Fisetin Ameliorated Senescence-Associated Secretory Phenotype From Senescent Alveolar Epithelial Cells and Reduced Collagen Deposition in Fibroblasts

**FIGURE 6:**
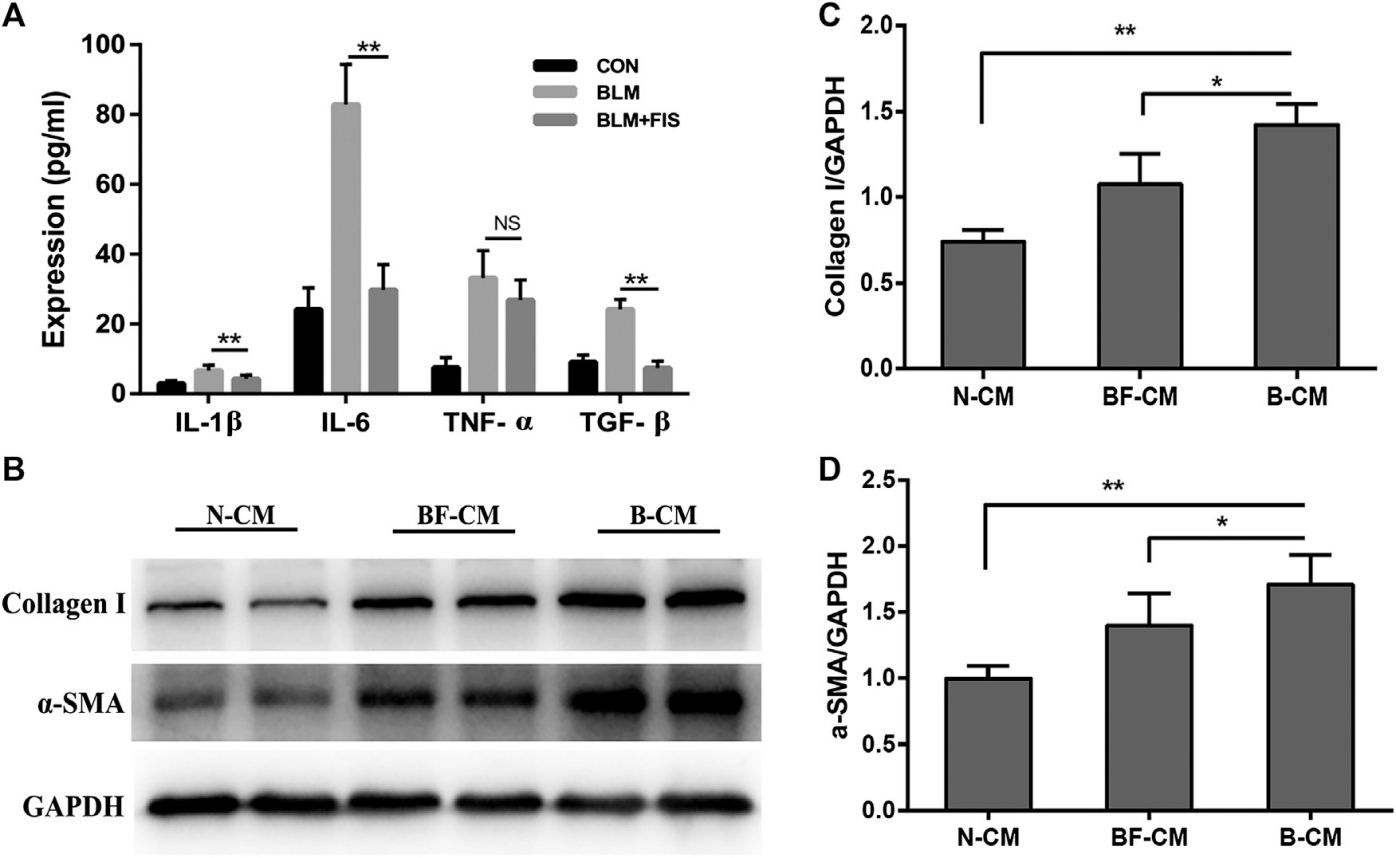
FIS ameliorated SASP from senescent A549 cells and reduced collagen deposition in HELF cells. After treatment with or without 5 μg/ml of BLM and 10 μM FIS for 3 days, the culture supernatants of A549 cells were replaced by fresh culture mediums, and A549 cells were cultured for another 2 days. A549-derived conditioned mediums were collected to analyze cytokines and further culture HELF cells with one half of concentration for 3 days. The levels of IL-1β, IL-6, TGF-β, and TNF-α in conditioned mediums were detected by ELISA assay **(A)**. Protein expressions of collagen I and *α*-SMA in HELF cells were detected by Western blotting analysis **(B–D)**. N-CM, Normal A549-derived conditioned mediums. B-CM, BLM-induced A549-derived conditioned mediums. BF-CM, BLM and FIS co-stimulated A549-derived conditioned mediums. Data were expressed as mean ± SD (*n* ≥ 3). ^*^ *p* < 0.05, ^**^ *p* < 0.01. NS, non-significant.

Several widely-used cytokines, IL-1β, TNF-α, IL-6 and TGF-β were detected to reflect SASP from A549 cells in this study (Aoshiba et al., 2013; Tian et al., 2019). In Figure 6A, the levels of IL-1β, TNF-α, TGF-β, and IL-6 in the BLM-induced A549-derived conditioned mediums (B-CM) were higher than those in the normal A549-derived conditioned mediums (N-CM). All the above inflammatory cytokines, except for TNF-α, were significantly ameliorated in the BLM and FIS co-stimulated A549-derived conditioned mediums (BF-CM). As shown in Figures 6B–D, exposure of HELF cells to conditioned mediums for 3 days, increased protein expressions of collagen 1 and *α*-SMA were detected in B-CM stimulated HELF cells as compared to N-CM cultured HELF cells, whereas BF-CM obviously decreased the protein expressions of collagen 1 and *α*-SMA in HELF cells as compared to B-CM. These results suggest that FIS can ameliorate SASP from senescent alveolar epithelial cells, and therefore reduce transdifferentiation of fibroblasts to myofibroblasts as well as collagen deposition in fibroblasts.

## Discussion

PF is a serious threat to human health, however, for many patients, effective treatment options are still lacking. Therefore, novel therapeutic strategies are urgently required. FIS is a dietary flavonoid, the average daily intake of naturally occurring FIS in Japan is about 0.4 mg/day (Kimira et al., 1998; Arai et al., 2000). Mounting evidence has indicated that FIS has potential to prevent or treat a variety of chronic diseases by acting as an anti-inflammatory, anti-oxidant, anti-aging and anti-fibrotic agent (Pal et al., 2016; Choi et al., 2020). However, the role of FIS in PF remains unclear.

In the present study, FIS treatment significantly improved BLM-induced weight loss and lung pathological changes in mice. Additionally, FIS notably decreased the levels of collagen 1 and *α*-SMA as well as HYP contents in fibrotic lung tissues, indicating that FIS reduced the accumulation of ECM. TGF-β/Smads signaling appears to be critical in pulmonary fibrosis, and Smad3’s key role in TGF-β-induced deposition of ECM has been widely recognized (Dobaczewski et al., 2010). In this study, we also confirmed that FIS remarkably inhibited the expression of TGF-β and Smad3 phosphorylation in the BLM-induced PF. Although FIS did not demonstrate survival benefits, it showed a trend in favour of a reduction in mortality. These results suggest that FIS has protective effect on PF. Previous studies have shown that FIS supplementation prevents hepatic fibrosis by suppressing the gene expressions of COL1, MMP2, MMP3, and MMP9 and collagen accumulation (Choi et al., 2020). FIS treatment markedly reduces the expression of fibrosis-related genes and inhibits myocardial fibrosis by inactivating TGF-β/Smads signaling (Liu et al., 2019; Hu et al., 2020). More importantly, no side effects of FIS have been reported, even when given at high doses (Maher, 2015).

IPF is an aging-associated disease. In general, the number of senescent cells increases with age, however, cellular senescence is not a universal property in aged tissues, and senescence is not exactly equivalent to aging (Kuwano et al., 2016). In the lung, approximately 6.7% and 19% of senescent cells are detected in young and old mice, respectively; however, there are no obvious differences in the eye, skeletal muscle and heart (Wang et al., 2018). Accelerated cellular senescence is now considered as an important driving mechanism for IPF (Minagawa et al., 2011; Kuwano et al., 2016; Barnes et al., 2019). Although increased senescence has been identified in both alveolar epithelial cells and fibroblasts in the lung tissues of IPF patients, alveolar epithelial cells are the major target of BLM-induced senescence in mice, while fibroblasts are not affected to a great extent (Aoshiba et al., 2013; Lehmann et al., 2017). Interestingly, the role of senescent fibroblasts in PF is conflicting, since both an anti-fibrotic role (Cui et al., 2017) and a detrimental role (Schafer et al., 2017) have been reported. However, almost all reports indicate that senescent alveolar epithelium cells, as a major senescent cell type in IPF, are detrimental in PF patients (Lehmann et al., 2017; Tian et al., 2019).

Although A549 cells are a lung cancer cell lines, their response to BLM-induced senescence is not significantly different from that of normal alveolar epithelial cells (Aoshiba et al., 2003). Besides, primary alveolar epithelial cells are difficult to obtain and maintain *in vitro*. Therefore, A549 cells were used as an alveolar epithelial cell model in this study. In line with previous studies (Aoshiba et al., 2003; Tian et al., 2019), increased senescence was found in alveolar epithelium cells of murine fibrotic lungs and cultured A549 cells treated with BLM, while FIS administration apparently reduced senescent cell burden along with decreased fibrotic markers. Therefore, the anti-fibrotic effects of FIS is at least partly related to its senolytic activity. It’s not surprising, quercetin, a flavonoid family member, has shown anti-fibrotic effect by reducing senescent cell burden in PF, while FIS has more stronger senotherapeutic activity than quercetin (Yousefzadeh et al., 2018; Hohmann et al., 2019).

Different from apoptotic cells, senescent epithelial cells have metabolic activity and may secrete multiple growth factors, cytokines, and proteases, known as SASP, leading to low-grade chronic inflammation, which further initiates senescence and abnormal epithelial cell-fibroblast interactions, promotes the transformation of fibroblasts into myofibroblasts and considerable ECM accumulation (Aoshiba et al., 2013; Tian et al., 2019). Multiple inflammatory cytokines in the supernatant of senescent fibrotic alveolar epithelial cells were up regulated 1.5 times by analysis using mass spectrometry proteomics (Lehmann et al., 2017). FIS can inhibit the release of various inflammatory cytokines, such as IL-1β, TNF-α, IL-6 and NF-κB, which are also considered to be SASP components (Gupta et al., 2014). As expected, FIS treatment obviously decreased levels of inflammatory cytokines (TNF-α, IL-6 and IL-1β) and pro-fibrosis factor (TGF-β) in the BLAF of BLM-induced mice, exerted its anti-inflammatory effect.

As we all know, one important anti-fibrosis pathway is to effectively inhibit the synthesis of collagen or increase its degradation. Collagen, mainly secreted by myofibroblasts, is the most important part of ECM, while *α*-SMA is a specific biomarker of fibroblast to myofibroblast transdifferentiation (Yang J. Y. et al., 2019). *In vitro* study, we also found that FIS suppressed the secretion of inflammatory cytokines by senescent alveolar epithelium cells, followed by reduced transdifferentiation of fibroblasts to myofibroblasts and collagen deposition in fibroblasts. These results suggest that FIS treatment can improve aberrant inflammatory microenvironment by reducing alveolar epithelial cell senescence and accompanying SASP, which is beneficial to maintain normal function of fibroblasts and reduce ECM accumulation.

Energy metabolism regulates tissue repair and remodeling responses. As a pivotal regulator of energy metabolism, AMPK plays important roles in the process of aging and fibrogenesis (Salminen and Kaarniranta, 2012; Jiang et al., 2017). The lack of AMPK activity with aging can provoke many age-associated diseases, including IPF. AMPK activation is significantly decreased in regions of active fibrosis of IPF patients and mouse model. The activators of AMPK can inhibit the expression of *α*-SMA, collagen and fibronectin in fibroblasts stimulated with TGF-β, and reverse established lung fibrosis in an AMPK-dependent manner in BLM-induced mice (Rangarajan et al., 2018). NF-κB is the primary regulator of the appearance of SASP, the activation of AMPK can inhibit NF-κB signaling and inflammation (Salminen et al., 2011). Some reports have established the role of FIS in regulating AMPK/NF-κB signaling pathways (Liu et al., 2019; Yang W. et al., 2019). Consistently, our results revealed that FIS could activate AMPK and inhibit NF-κB activity both *in vivo* and *in vitro*, whereas such changes were partly blocked by AMPK inhibitor, accompanied by elevated senescence markers. These results indicate that AMPK/NF-κB signal pathway is involved in the anti-aging and fibrotic effects of FIS in PF.

## Conclusion

Taken together, FIS can alleviate the development of BLM-induced pulmonary fibrosis, which is related to the inhibition of TGF-β/Smad3 signaling and the reduction of alveolar epithelium cell senescence by regulating AMPK/NF-κB signaling pathway. FIS may be a promising candidate for patients with PF.

## Data Availability Statement

The raw data supporting the conclusions of this article will be made available by the authors, without undue reservation.

## Ethics Statement

The animal study was reviewed and approved by the Committee on the Ethics of Animal Experiments of Sichuan University.

## Author Contributions

LZ, XT, JH, and HF conceived the experiment, LZ, JH, XT, and MW conducted the experiments, LZ, DW, and SZ analyzed the results, and LZ and SL contributed to the manuscript writing. All of the authors reviewed the manuscript.

## Funding

This study was supported by National Key R&D Program of China (2017YFC1309703), China Postdoctoral Science Foundation (2020M673259), 1·3·5 project for disciplines of excellence–Clinical Research Incubation Project, West China Hospital, Sichuan University (2019HXFH008) and Post-Doctor Research Project, West China Hospital, Sichuan University (2020HXBH013).

## Conflict of Interest

The authors declare that the research was conducted in the absence of any commercial or financial relationships that could be construed as a potential conflict of interest.
